# Clinicopathologic and Molecular Analysis of Inflammatory Pseudotumor-Like Follicular/Fibroblastic Dendritic Cell Sarcoma: A Case Report and Review of Literature

**DOI:** 10.5146/tjpath.2021.01523

**Published:** 2021-09-15

**Authors:** Bryan Morales-Vargas, Kristin Deeb, Deniz Peker

**Affiliations:** Department of Pathology and Laboratory Medicine, Emory University School of Medicine, Georgia, USA

**Keywords:** Inflammatory pseudotumor-like follicular/fibroblastic dendritic cell sarcoma, Splenic tumor, BCOR1L1, JAK2

## Abstract

Inflammatory pseudotumor-like follicular/fibroblastic dendritic cell (FDC/FRC) sarcoma is an extremely rare neoplasm of the spleen associated with EBV and characterized by spindle cell morphology, dense mixed chronic inflammatory background, and a broad immunophenotypic profile often causing a diagnostic challenge for pathologists. The molecular features of FDC/FRC sarcoma are largely unknown due to a lack of comprehensive data. Here we present the results of next-generation sequencing and Single Nucleotide Polymorphism Copy Number array analysis in a case of FDC/FRC and review the literature.

## INTRODUCTION

Inflammatory pseudotumor-like follicular/fibroblastic dendritic cell (FDC/FRC) sarcoma, a subgroup of follicular dendritic cell sarcoma (FDC), is rare neoplasm that often occurs in the spleen (1–15) . FDC/FRC sarcoma can involve other anatomical sites including the liver (13,16–23), colon (24,25), and peripancreas (17,20). The tumor has a predilection for young to middle-age adults and may present with various symptoms including fever, abdominal pain, sweats, fatigue (predominantly in women), and more often splenic solitary lesions (5,6,9). FDC/FRC sarcoma is a lesion of neoplastic spindled cells with often follicular dendritic cell differentiation and over 90% of the cases are associated with EBV infection (9). Here, we discuss the clinicopathologic and molecular features of a localized splenic FDC/FRC sarcoma in an older age female and review the English-language literature.

## CASE REPORT

A 66-year-old female patient presented with a two-month history of left upper quadrant pain. She had no relevant clinical history and denied systemic symptoms including fever, weight loss, and night sweats. Physical exam was overall unremarkable. Imaging studies revealed a well-defined splenic nodule measuring 4.5 cm on ultrasound and 5.0 cm on computerized tomography (CT), which was not present on a prior CT performed in 2017. There were no suspicious lymph node enlargement, hepatomegaly, or other organ abnormalities on abdominal computerized tomography. She underwent elective splenectomy.

### Histomorphologic, Immunophenotypic, and Molecular Analysis

**Figure 1:**
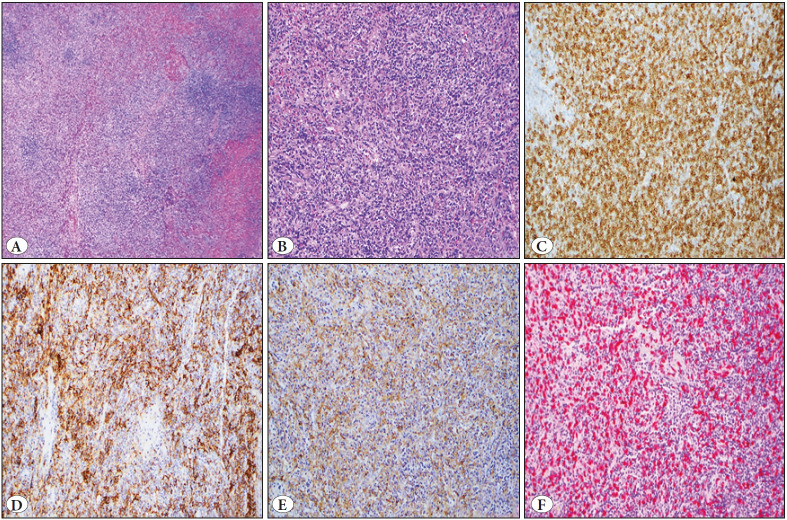
**A)** Splenic parenchyma with partially altered architecture (H&E; x20) **B)** by the spindled cells in the background of chronic inflammatory cells (H&E; x200). The spindled cells show expression of **C)** CD35 (IHC; x200), **D)** CD23 (IHC; x200), **E)** SMA (IHC; x200), with numerous cells positive for **F)** EBER (ISH; x200).

Macroscopic examination revealed a 130 mg spleen containing a well-circumscribed, moderately firm, red-pink mass. The hematoxylin and eosin sections demonstrated splenic parenchyma with a lesion that resembled splenic parenchyma with white and red pulp structures without overt morphologic atypia (Figure 1). Also noted were somewhat spindled histiocytoid cell clusters that had a moderate amount of pale cytoplasm, oval vesicular nuclei, inconspicuous nucleoli, delicate fibrous bands, and increased vascularity (Figure 1). In addition, there was a polymorphous proliferation of plasma cells and small lymphocytes admixed in the background. Ancillary immunohistochemical studies were performed on formalin-fixed, paraffin-embedded (FFPE) tissue sections following routine procedures for CD20, CD3, CD5, smooth muscle actin (SMA), CD20, CD21, CD23, CD68, BLC6, IgG, IgG4, PAX-5 AE1/AE3, and Ki-67. Epstein Barr virus encoded small RNAs (EBER) in situ hybridization was performed with immunohistochemistry/EBER (please see Table 1 for additional antibody information). CD3, CD5, and CD43 were expressed in the background T-cell population while CD20 and PAX-5 highlighted the B-cells forming follicles in the white pulp with partial BCL6 expression. Atypical spindled cells were positive for CD35 (Figure 1), CD23 (Figure 1), and CD21 (partial) as well as SMA (Figure 1), consistent with a follicular dendritic cell and myofibroblastic origin, respectively. EBV-encoded small RNA was extensively positive using in situ hybridization (EBER) (Figure 1).

**Table 1:** Antibodies used in this study

**Antigen**	**Clone**	**Dilution**	**Source**	**Platform used**
CD3	LN10	Ready-to-use	Leica Microsystems, Buffalo Groove, IL, USA	Bond Automated IHC Stainer
CD5	4C7	Ready-to-use	Leica Microsystems, Buffalo Groove, IL, USA	Bond Automated IHC Stainer
CD20	L26	Ready-to-use	Leica Microsystems, Buffalo Groove, IL, USA	Bond Automated IHC Stainer
CD21	2G9	Ready-to-use	Leica Microsystems, Buffalo Groove, IL, USA	Bond Automated IHC Stainer
CD23	1B12	Ready-to-use	Leica Microsystems, Buffalo Groove, IL, USA	Bond Automated IHC Stainer
CD68	KP1	x 3200	Dako Cytomation, Carpinteria, CA, USA	Bond Automated IHC Stainer
BCL6	LN22	Ready-to-use	Leica Microsystems, Buffalo Groove, IL, USA	Bond Automated IHC Stainer
IgG		x 80,000	Dako Cytomation, Carpinteria, CA, USA	Bond Automated IHC Stainer
IgG4	HP6025	x 8,000	The Binding Site, Birmingham, United Kingdom	Bond Automated IHC Stainer
EBER	Leica Probe	Ready-to-use	Leica Microsystems, Buffalo Groove, IL, USA	Bond Automated IHC Stainer
PAX-5	24	x 2	Cell Marque, Rocklin, CA, USA	Bond Automated IHC Stainer
Smooth muscle actin	1A4	x 1600	Dako Cytomation, Carpinteria, CA, USA	Bond Automated IHC Stainer
Ki-67	MIB-1	x 80	Dako Cytomation, Carpinteria, CA, USA	Bond Automated IHC Stainer
AE1/AE3	AE1/AE3	x 200	Dako Cytomation, Carpinteria, CA, USA	Bond Automated IHC Stainer

**Figure 2:**
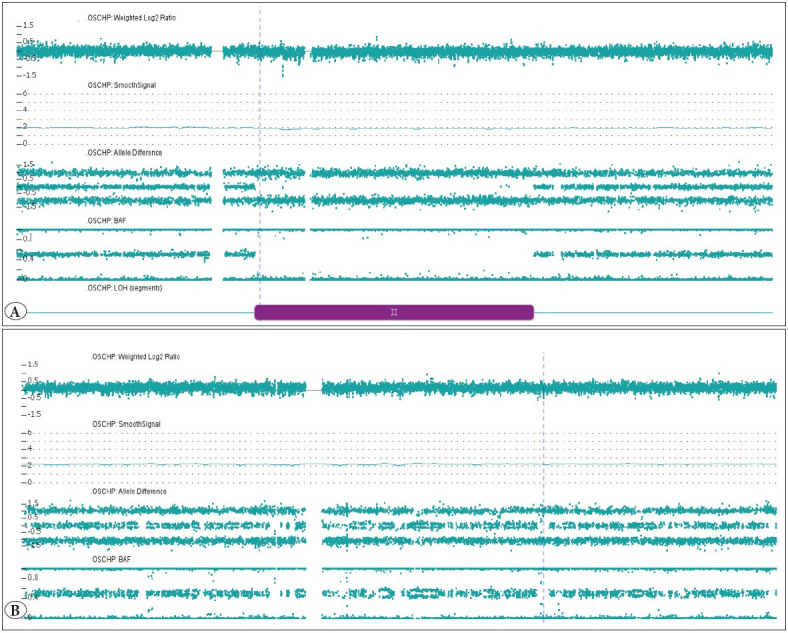
SNP-CN array analysis of chromosome 5 and chromosome X. **A)** Approximately 66.8-Mb (megabase) stretch of copy neutral loss of heterozygosity (CN-LOH) was detected interstitially on the long arm of chromosome 5. **B)** A clonal low-level loss of the entire X chromosome was detected in the splenic specimen.

Single Nucleotide Polymorphism Copy Number (SNP-CN) array analysis using the Thermo Fisher OncoScan FFPE Assay Kit (Thermo Fisher Scientific, Waltham, MA) was performed according to the manufacturer’s directions and revealed a copy neutral loss of heterozygosity of 5q in 100% of the specimens, which was considered constitutional. Also detected was a gain of the X chromosome that was clonal and the clinical significance of this finding was unclear (Figure 2). To better characterize this tumor, somatic mutation detection by next-generation sequencing was performed (Myeloid 75-gene targeted sequencing panel). Two variants were detected by the Archer® VariantPlex Myeloid 75-gene panel *BCORL1 *c.3463C>A (p.P1155T) in approximately 56% of the alleles and *JAK2 *c.2852T>C (p.I951T) in approximately 47% of the alleles. These two variants are observed as germline variants in a minor proportion of the population with BCORL1 seen in 6 alleles and JAK2 seen in 10 alleles (both with minor allele frequencies of approximately 3.6x10-5) (26). *In silico *algorithms predict the variants to be tolerated/benign (provean.jcvi.org). Therefore, given the information currently available, the clinical significance of these variants is uncertain at best. Polymerase chain reaction did not detect rearrangement of the T-cell receptor or *IgH*.

A diagnosis of inflammatory pseudotumor-like follicular/fibroblastic dendritic cell tumor (FDC/FRC) was rendered. The patient was followed up for 6 months after the surgery and no recurrent disease was identified. She remains in remission to our knowledge.

## DISCUSSION

FDC/FRC is a rare subtype of FDCS with a favorable clinical course, and surgical treatment alone is often the therapeutic approach. A slight female predilection was observed with a male to female ratio of 0.6. The tumor affects mostly the adult population with the age varying between 19 and 79 years. One third of the patients were asymptomatic (n=21) while the remaining patients presented with various symptoms including abdominal pain, and gastrointestinal and other systemic symptoms. EBV positivity in tumor cells was consistently present. At least one FDC marker was positive in nearly all patients except for one case (15), and SMA was expressed in the majority of the cases.

The histomorphologic and immunophenotypic features of FDC/FRC resemble other spindled cell tumors that are more aggressive and potentially require more aggressive treatments. Therefore, it is crucial to perform a thorough diagnostic work-up to render an accurate diagnosis. FDC/FRC sarcoma harbors mesenchymal and inflammatory components and a wide range of immunophenotypic profiles causing a diagnostic challenge in differentiating it from other spindle cell tumors of the spleen including follicular dendritic cell sarcoma, interdigitating dendritic cell sarcoma arising in or involving the spleen, fibroblastic reticular cell tumor, and reactive processes such as splenic inflammatory pseudotumor also known as inflammatory myofibroblastic tumor (Table 2). Ge et al. have authored a review manuscript and described the histomorphologic characteristics of FDC/FRC sarcoma in detail, including proliferation of neoplastic spindled cells with varying degree of nuclear atypia, a vesicular chromatin pattern, and conspicuous nucleoli in a background of lymphoplasmacytic infiltrate that may be accompanied by eosinophils, a granulomatous reaction, necrosis, and hemorrhage (9). The neoplastic cells are often of follicular dendritic cell origin with expression of CD21, CD23, or CD35, similar to follicular dendritic cell sarcoma (FDCS). However, although the spindled cell morphology and expression of dendritic cell markers are often shared by these two tumors, there are several differences: FDC/FRC sarcoma is often an isolated lesion and almost exclusively associated with EBV. FDC may occur in multiple sites and, in addition to FDC markers, Clusterin, a glycoprotein associated with apoptosis, is frequently expressed in FDCS (27), although not reported in FDC/FRC sarcoma. On the other hand, fibroblastic reticular cell differentiation and SMA expression are not commonly observed in FDCS (28). The differentiation between FDC/FRC sarcoma and FDCs is crucial given the biological behavior of inflammatory FDC/FRC sarcoma is more indolent than a true intra-abdominal FDC. Interdigitating dendritic cell sarcoma (IDCS) is another dendritic cell tumor characterized by neoplastic proliferation of spindled to ovoid cells arranged in fascicles or whorls or in a storiform pattern (1). Tumor cells are consistently positive with S100 and Vimentin, which are mostly negative in FDC/FRC sarcoma. IDCS has an aggressive clinical course in the majority of the cases. Fibroblastic reticular cell tumor (FRCT) is a rare neoplasm that can be seen in the spleen. FRCT has a similar histomorphology to other dendritic cell tumors with a rather distinct immunophenotype including expression of SMA, desmin, and CD68 as well as cytokeratin, which separates it from the others. Our case was also negative with AE1/AE3. Splenic inflammatory pseudotumor (IPT) is a rare benign reactive process resulting from an infectious, autoimmune or reparative process, with subsets expressing EBV (29). EBV+ IPT often has a similar histomorphology to FDC/FRC sarcoma including isolated nodules formed by a bland spindled myofibroblastic proliferation and a polymorphic inflammatory infiltrate that can be accompanied by hemorrhage. The myofibroblasts are characteristically positive for SMA, Vimentin and, in subsets, CD68 without expression of FDC markers (30).

**Table 2:** Clinicopathologic and immunophenotypic features of spindled cell tumors of the spleen

	**Demographics**	**Most common localization**	**Histology**	**Immunophenotype**	**Prognosis**
**FDC/FRC**	Adult/Female predilection	Spleen, liver, gastrointestinal tract	Spindled cells, bland to pleomorphic morphology, dense background lymphoplasmacytic infiltrate, necrosis and hemorrhage	CD21+, CD23+, CD35+, SMA+, EBV+	Good prognosis, local recurrence
**FDC**	Adult/ no sex predilection	Lymph node, tonsil, gastrointestinal tact soft tissue	Spindled to ovoid cells, forming whorls, fascicles, storiform arrays, often bland cytology, less dense lymphocytic infiltrate	CD21+, CD23+, CD35+, Fascin+, Clusterin+, CD68+	Worse prognosis; large size, cytologic atypia, necrosis, and increased mitotic activity
**IDCS**	Adult/Male predilection	Lymph node, skin, soft tissue	Round to spindled cells forming sheets of fascicles, whorls or arranged in storiform pattern. Necrosis not present.	S100+, Vimentin+, Fascin+. No FDC marker expression	Often aggressive clinical course
**FRCT**	Adult/Male predilection	Lymph node, spleen, soft tissue	Similar to FDC	SMA+, Desmin+, Cytokeratin+, CD68+	Variable (limited data)
**EBV+ IPT**	Adult/Female predilection	Spleen	Similar to FDC	SMA+, Vimentin, CD68, EBV+. No FDC marker expression	Good prognosis

**FDC/FRC:** Inflammatory pseudotumor-like follicular/fibroblastic dendritic cell; **FDC:** Follicular dendritic cell sarcoma; **IDCS:** Interdigitating dendritic cell sarcoma; **FRCT:** Fibroblastic retocular cell tumor; EBV-positive **IPT:** EBV-positive inflammatory pseudotumor.

The molecular pathogenesis of the disease is poorly understood. Gain of chromosome X was detected by SNP array in our case and was considered clonal; however, the association of such an anomaly with FDC/FRC sarcoma has not been reported to our knowledge. Gain of the X chromosome in the current case may suggest chromosome X mosaicism, which is usually age-related and preferentially affects the inactivated X chromosome (31). The existing data from cancer genomes indicate that the female X chromosome, particularly inactive X, usually has a higher mutation burden of point mutations and that this is a frequent event in cancer (32). Bruehl et al. recently reported the results of comprehensive mutation analysis using NGS in two cases of FDC/FRC sarcoma and found no variants of strong or potential clinical significance (33). The results of NGS in the current case were similar; variants were detected in *BCORL1 *and *JAK2*. However, the clinical significance of *BCORL1* and *JAK2*, if any, is uncertain given their presence in a very minor fraction of the population database. Furthermore, whether these germline variants have susceptibility or predispose to hematologic malignancies are unknown at this time. *BCORL1 and JAK2 *gene alterations have been demonstrated in various tumors including myeloid neoplasms. Additionally, *BCORL1 *has been shown in medulloblastoma, retinoblastoma, and uterine sarcoma (34,35) and might potentially be a pathogenic gene in the development of FDC/FRC. Although these variants may represent heterozygous germline sequence variants, the clinical significance of these variants in the context of this case is undetermined at this time and larger scale case series and studies are warranted to better understand the biology and clinical implications of this rare disease.
